# Selecting measures of visual function to classify diabetic retinopathy status: a cross-sectional study

**DOI:** 10.1136/bmjophth-2025-002536

**Published:** 2026-01-20

**Authors:** David M Wright, Usha Chakravarthy, Radha Das, Katie W Graham, Timos T Naskas, Tunde Peto, Ruth E Hogg

**Affiliations:** 1 Centre for Public Health, Queen’s University Belfast, Belfast, UK

**Keywords:** Retina, Vision

## Abstract

**Aim:**

To identify combinations of up to three visual function tests with the best performance for classifying diabetic retinopathy (DR) severity stage. To describe in detail the measurements from a comprehensive set of visual function tests.

**Methods:**

1901 eyes (1032 participants) underwent nine visual function tests. Fundus, ultra-widefield and optical coherence tomography images were graded for DR and diabetic macular oedema (DMO). Three classification tasks were set: (1) distinguishing diabetes mellitus (DM) no DR from healthy with no DM, (2) DR no DMO from DM no DR and (3) DR with DMO from DR no DMO. Ensemble machine learning models for all one-way, two-way and three-way combinations of visual function variables were compared using area under the curve (AUC).

**Results:**

The top 30 models for each task achieved high accuracy, with AUC ≥0.94. For task 1, 17/30 top models contained distance visual acuity. Pelli-Robson contrast sensitivity and low luminance visual acuity also featured highly. For task 2, 19/30 models contained mesopic microperimetry. Near visual acuity, matrix microperimetry and reading index featured highly. For task 3, 17/30 models contained distance visual acuity. Smith-Kettlewell low luminance near visual acuity and near visual acuity featured highly. In a subset of eyes where perimetry was not performed, reading index featured in 22, 21 and 22 of the top models for tasks 1, 2 and 3 respectively.

**Conclusions:**

These findings will enable researchers and those planning clinical trials to select the best combination of visual function tests for distinguishing stages of diabetic eye disease.

**WHAT IS ALREADY KNOWN ON THIS TOPIC**

- A battery of nine visual function tests can be combined using machine learning models to categorise status of diabetic eye disease with high accuracy. We aimed to determine whether fewer tests (three or fewer) could achieve the same performance.

**WHAT THIS STUDY ADDS**

- Machine learning models categorised status of diabetic eye disease with high accuracy using just age, sex and at most three measures of visual function.
- Selected tests varied depending on the disease stage to be distinguished.

**HOW THIS STUDY MIGHT AFFECT RESEARCH, PRACTICE OR POLICY**

- These findings provide a comprehensive reference for those planning clinical trials or diagnostic test accuracy studies with visual function as an outcome.
- They will enable researchers to select the best and most feasible combination of tests for distinguishing each stage of diabetic eye disease.

## Introduction

 Diabetes mellitus (DM) is a major cause of vision loss through development of diabetic retinopathy (DR) and diabetic macular oedema (DMO). Anatomical signs of DR may take years to develop, but early detection and treatment are crucial.^1^

In other chronic eye diseases such as glaucoma, insights into pathophysiology have been gained through visual function testing, and some functional tests have now been Food and Drug Administration (FDA) approved as clinical trial endpoints.^2^ Various groups, including ourselves, have highlighted that functional decline can occur prior to standard clinical retinal signs.^3 4^

Identifying new functional endpoints for diabetic eye disease would be similarly valuable.^5^ Associations between diabetic eye disease, especially late-stage disease, and reduced visual function have been established across a wide range of measures including contrast sensitivity, frequency doubling perimetry mean deviation, increased photostress recovery time and dark adaptation speed.^5 6^

However, much detail is lacking that would be required for effective trial design. Functional studies are typically small, and careful selection of ‘normal’ or ‘control’ subjects as comparators may not have captured the range of variation expected in a heterogeneous DM population. Consequently, tests may not generalise well, and providing larger and more diverse normative datasets is a priority to enable more accurate effect and sample size estimation.^2^

Visual function is multidimensional and individual tests may only address particular aspects of the visual system. For example, some focus on rod or cone mediated pathways, whereas others are more integrative (eg, reading speed). Therefore, a combination of tests may provide additional information to characterise disease stages and produce composite endpoints.

We recently demonstrated a machine learning (ML) approach to combine nine visual function tests that substantially improved classification performance compared with a more conventional statistical analysis.^7^ Such a broad battery of tests is too costly in terms of time and money for most trials and clinical settings, but identifying subsets of functional tests that discriminate best between each disease stage would be valuable to inform trial design and clinical practice.

Our aims were to:

1. Describe in detail the measurements resulting from a rich visual function assessment conducted on over 1000 individuals, with diabetic eye disease status ranging from none to DMO.
2. Establish which combinations of visual function tests have the best discriminative performance for classifying DR severity stage.

For aim 2, three classification tasks reflected clinical situations in which determining DR status rapidly using non-invasive methods would be useful:

- Distinguishing the retinae of those with DM and no DR from retinae of normal individuals without DM (DM no DR vs no DM).
- Distinguishing those with DR from those with DM and no DR. This evaluates whether clinically relevant DR features (associated with worse long-term outcomes) are detectable within a DM population by measuring visual function (DR no DMO vs DM no DR).
- Within the DR population, distinguishing those with DMO from those without DMO (DR with DMO vs DR no DMO).

We restricted analysis to combinations of three or fewer visual function tests as studies with larger numbers of tests are unlikely to be feasible. Model performance was benchmarked against our previously published study which used all nine tests simultaneously and achieved area under the curve (AUC) values of 1.00, 1.00 and 0.93 for tasks 1, 2 and 3, respectively.^7^

## Methods

### Data Ccollection

The majority of participants were drawn from the Northern Ireland Sensory Ageing Study (NISA) (https://clinicaltrials.gov/ct2/show/NCT02788695) (n=881), an add-on to the Northern Ireland Cohort for the Longitudinal Study of Ageing (NICOLA).^8^ NICOLA participants with DM (either self-reported or HbA1c ≥48 mmol/mol (6.5%)), those with early or intermediate age-related macular degeneration (AMD) and those with no retinal diseases and no DM were called for an additional study visit.

Additional participants with confirmed diabetes diagnoses were recruited from diabetes clinics (n=150), together with healthy volunteers (n=91) with no history of eye disease aged under 50. These were a convenience sample with a similar age distribution to the younger clinical participants, comprising university employees, their friends or family or participants recruited via advertising. Patients or the public were not involved in the design, or conduct, or reporting, or dissemination plans of our research.

The overall cohort comprised 2244 eyes measured across 1122 participants recruited between 2014 and 2018. Methods have been described in detail previously^7^ but in brief, we concentrated on diabetic eye disease, excluding 343 eyes with other conditions affecting visual function such as AMD or glaucoma. The analysis cohort comprised 1901 eyes from 1032 individuals. About 61% of eyes were from females, median age was 64.

#### Retinal imaging

Participants underwent retinal imaging after pharmacologic dilation with tropicamide 1%: fundus colour photography (CX-1 digital fundus camera; Canon, USA), colour ultra-widefield imaging centred on the fovea (Optomap Panoramic 200Tx scanning laser ophthalmoscope; Optos, UK) and Spectralis spectral domain-optical coherence tomography (SD-OCT; Heidelberg Engineering, Germany). DR status was established following detailed grading of images. Ultra-widefield capture on colour allowed scrutiny of the far retinal periphery, where retinopathy can be seen in a proportion of eyes without features of DR elsewhere. Presence of DMO was determined on SD-OCT.

#### DR classification

Disc and macula colour images and images obtained by ultra-widefield imaging were assessed for features of DR in the central and peripheral retina and then staged using the national screening for DR system for England and Wales into four levels: none (R0), background (R1), preproliferative (R2) and proliferative (R3).^9^ Participants over 50 years of age and all patients with diabetes were invited for blood sampling to measure plasma HbA1c. Participants with no record of diabetes were classified as having diabetes if their HbA1c was ≥48 mmol/mol (6.5%).

#### Visual function tests

Nine visual function tests were performed after full refraction by an experienced optometrist. For perimetry-based tests, the eye with better BCVA (best-corrected visual acuity) was selected for the study, choosing at random if both were eligible. Tests were chosen to cover the breadth of functional deficits previously reported in diabetes with an emphasis on methodologies that could be easily applied in a clinical setting if found to be predictive.

Full test details are outlined in the online supplemental material and elsewhere.^7^ The following tests were performed: (1) distance visual acuity (DVA); (2) near visual acuity (NVA); (3) reading index^10 11^; (4) distance low-luminance visual acuity; (5) near low-luminance visual acuity^12^; (6) contrast sensitivity; (7) the Moorfields acuity chart^13^; (8) frequency doubling technology perimetry on central visual field; and (9) microperimetry macular integrity assessment.^14^ Some of the tests (eg, microperimetry) produced multiple output variables, so there were 12 visual function variables in total. Where a test contributed multiple variables, these were presented together for modelling in their original form when that test was selected.

### Visual function distributions

Eyes were classified into four groups: no DM; DM no DR; DR no DMO; DR with DMO. Distribution of visual function variables by group was described with statistics typically used in power and sample size calculations: n, mean, SD and 2.5%, 25%, 50%, 75% and 97.5% quantiles.

### Selection of visual function tests

The three tasks were (1) no DM vs DM no DR, (2) DR no DMO vs DM no DR, (3) DR with DMO vs DR no DMO. We fitted three sets of ensemble ML models, one containing each visual function test singly, the second and third containing each two-way and three-way combination of tests, respectively (using all 9 tests, a total of 129 model fits for each task: 84 three-way, 36 two-way, 9 one-way combinations). Age and sex were included in all models. For each combination, instances with missing measurements for any of the selected tests were excluded, so analysis sets varied in size among combinations within reasonable limits (see ‘Analysis’ section below for full explanation).

We used a flexible data-driven approach, each model comprising a diverse suite of statistical submodels (learners) selected to cover a wide range of plausible functional forms (a SuperLearner). These comprised simple intercept-only models, regression-based models for correlated variables (ridge regression), variable selection (least absolute shrinkage and selection operator) and curvilinear relationships (polynomial splines). To model threshold-type associations and interactions among multiple variables, we included single-layer neural networks and tree-based methods (XGBoost, random forest and Bayesian additive regression trees).

For each task, each learner was fitted to 90% of the dataset and used to infer classifications for the remaining 10% (validation set). This was repeated 10 times, combining validation sets to give a complete set of classifications (ie, 10-fold crossvalidation).^15^ Individuals were used as the units of assignment for crossvalidation to prevent data leakage if one eye was used to train and the other eye to validate.

Submodel outputs were combined to predict probability of membership in the comparison class for that task (ie, Pr(DM no DR); Pr(DR no DMO); Pr(DR with DMO) for tasks 1–3, respectively). The final prediction for each eye was a linear combination of submodel predictions, weighted in a meta-learning step as outlined in Naimi and Balzer.^15^ The weights in the linear combination indicated the relative contribution of each learner to the final prediction (summing to 1). The overall algorithm (SuperLearner) achieves the best possible classification provided that one of the learners in the library approximates the true data-generating mechanism.^15^ Eyes with a probability >0.5 were labelled into the comparison class; others were labelled into the reference class (respectively: no DM; DM no DR; DR no DMO). Model performance was measured using AUC, area under the receiver operating characteristics curve and models were ranked by AUC for each task. To set the ensemble models in context, we also performed a more standard analysis, fitting each combination of tests using a main effects logistic regression.

### Analysis

The analysis cohort consisted of those with no DM and those with varying degrees of diabetic eye disease. The core battery of functional tests was applied in both eyes across all groups, but for logistical reasons, two of the more time-consuming tests, matrix perimetry and mesopic microperimetry, were restricted to the diabetes group and a sample of the no DM group. In most cases, perimetry was only performed on one ‘study eye,’ that with the best DVA. The Moorfields acuity chart was introduced to the test battery after sampling had begun, so not all eyes were tested with it.

To extract the maximum amount of information from the data, we conducted parallel analyses to quantify model performance in two contexts, structured to maximise data completeness in each and enable comparisons among models by ensuring they had similar data support. The first analysis included only those eyes that received the most time-consuming test, Matrix perimetry (‘all tests dataset’). In this analysis, measurements were largely complete (≥92%) for all tests and prediction tasks, with the exception of Moorfields chart acuity which had 58%, 69% and 71% completeness for tasks 1, 2 and 3, respectively. The second analysis was broader, based on seven visual function tests and included the full set of no DM eyes in addition to both the study and non-study eyes in the diabetes groups (‘no perimetry dataset’). The no perimetry dataset was thus a superset of the ‘all tests’ dataset. It had ≥89% completeness for all tests and prediction tasks except Moorfields chart acuity which had 76%, 71% and 70% for tasks 1, 2 and 3, respectively. Thus, within each analysis model, performance can be compared with confidence, with the possible exception of those including Moorfields chart acuity.

To assess the relative usefulness of each visual function test in predicting DM/DR status, we chose a simple metric, the number of times a test featured in the top 30 models. Acknowledging that ranking of test combinations is likely to be dataset specific, we reasoned that tests featuring in many combinations in the top 30 are more likely to carry relevant information than those that did not.

### Patient and public involvement

Patients or the public were not involved in the design, or conduct, or reporting, or dissemination plans of our research.

## Results

### Visual function distributions

Characteristics for both analysis sets are given in table 1. Age distributions of the analysis groups overlapped. The lowest proportion of females was in the ‘no DM’ group. Distributions of visual function measures by DM/DR status for the ‘all tests’ and no perimetry sets are summarised in electronic supplementary material (ESM) (online supplemental tables 1 and 2). In general, increasing severity of diabetic eye disease was associated with decreasing visual function. The only notable difference between sets was that visual function was slightly better in the ‘no DM’ group in the no perimetry set.

**Table 1:** Distribution of cohort characteristics by diabetes and retinopathy status

Set	Description	Level	No DM	DM no DR	DR no DMO	DR with DMO
All tests	Eyes		324 (100%)	116 (100%)	91 (100%)	52 (100%)
All tests	Age	Median (IQR)	60 (49, 66)	67 (58.8, 73.2)	66 (59, 71)	61.5 (57, 67)
All tests	Sex	Female	187 (57.7%)	77 (66.4%)	72 (79.1%)	38 (73.1%)
All tests	Sex	Male	137 (42.3%)	39 (33.6%)	19 (20.9%)	14 (26.9%)
No perimetry	Eyes		1317 (100%)	278 (100%)	216 (100%)	90 (100%)
No perimetry	Age	Median (IQR)	63 (56, 69)	67 (60, 73)	66 (59, 72)	62 (58, 67)
No perimetry	Sex	Female	731 (55.5%)	189 (68%)	167 (77.3%)	68 (75.6%)
No perimetry	Sex	Male	586 (44.5%)	89 (32%)	49 (22.7%)	22 (24.4%)

DM, diabetes mellitus; DMO, diabetic macular oedema; DR, diabetic retinopathy.

### Selection of visual function tests

**Figure 1:**
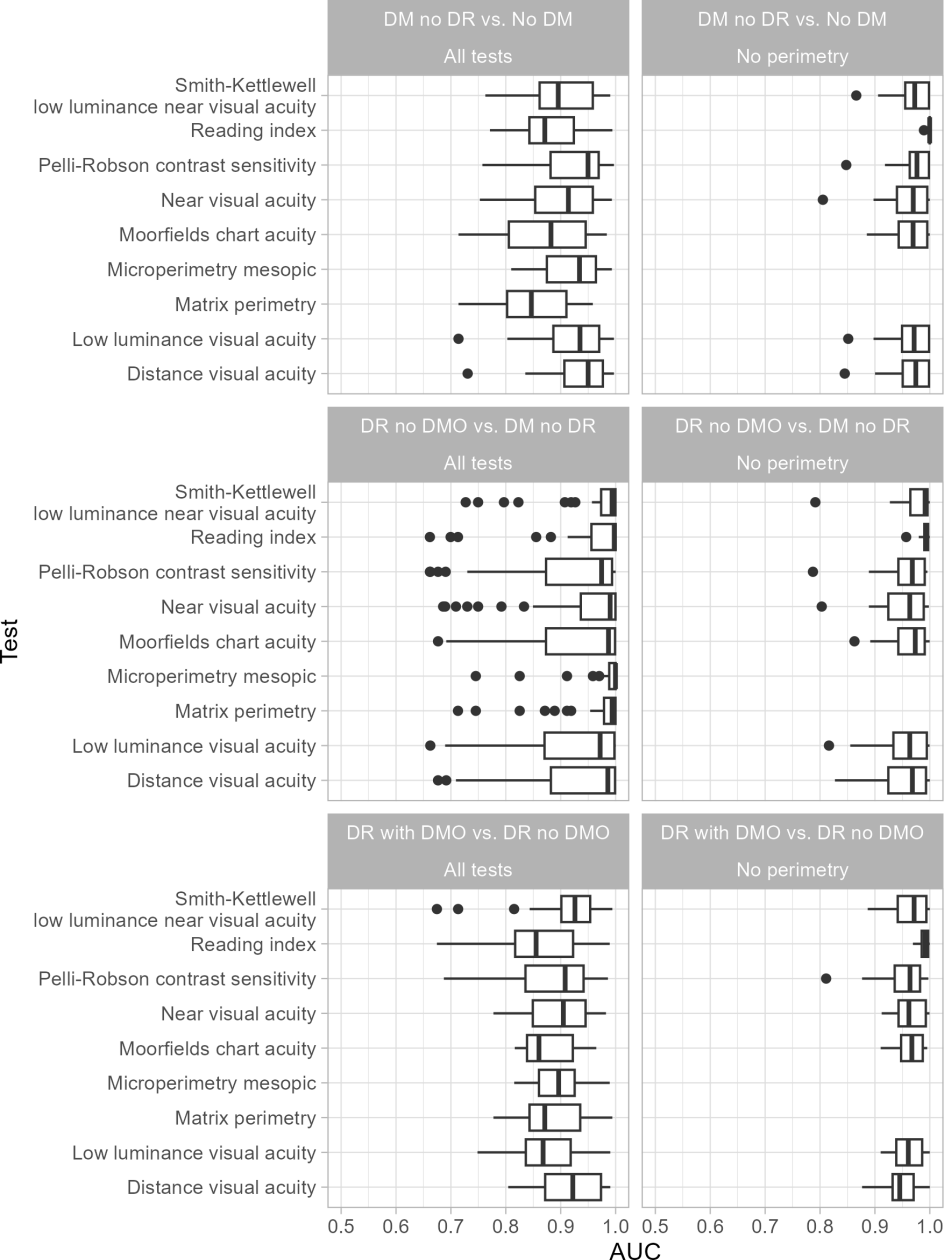
Distribution of model performance for each task by subgroup. AUC, area under the curve; DM, diabetes mellitus; DMO, diabetic macular oedema; DR, diabetic retinopathy.

Ensemble models varied widely in performance; AUC ranged from 0.66 (all tests, task 2) to maxima of 1.00 (all tasks). In comparison, our previously published models combining all nine visual function measures achieved AUCs of 1.00, 1.00 and 0.93 for tasks 1, 2 and 3, respectively.^7^ There was greater variation in performance within the ‘all tests’ dataset than the no perimetry dataset (figure 1).

Ensemble models matched or exceeded performance of logistic regression models fitted using the same data (ESM, online supplemental tables 3 and 4). Top-performing logistic models for each task had AUC of 0.80, 0.66 and 0.89 for tasks 1, 2 and 3, respectively, in the ‘all tests’ dataset. Performance was lower for each task in the no perimetry dataset.

Performance of the top-ranked models was similar across datasets and tasks, with AUC ≥0.99. All the models in the top 30 ranks had good performance with AUC ≥0.94.

Distinguishing eyes with DM no DR versus no DM (task 1), 17 of the top 30 models contained DVA. Low luminance visual acuity and Pelli-Robson contrast sensitivity were also concentrated in the top 30 models in the ‘all tests’ dataset (table 2). In the no perimetry dataset, the ranking changed such that reading index featured in 22 of the top 30 models.

**Table 2:** Number of times each visual function test featured in the top 30 classification models for each task

Task	Test	All tests	No perimetry
DM no DR vs No DM	Distance visual acuity	17	11
DM no DR vs No DM	Low luminance visual acuity	13	9
DM no DR vs No DM	Matrix perimetry	1	0
DM no DR vs No DM	Microperimetry mesopic	11	0
DM no DR vs No DM	Moorfields chart acuity	9	9
DM no DR vs No DM	Near visual acuity	10	8
DM no DR vs No DM	Pelli-Robson contrast sensitivity	13	12
DM no DR vs No DM	Reading index	5	22
DM no DR vs No DM	Smith-Kettlewell low luminance near visual acuity	9	10
DR no DMO vs DM no DR	Distance visual acuity	7	10
DR no DMO vs DM no DR	Low luminance visual acuity	6	9
DR no DMO vs DM no DR	Matrix perimetry	10	0
DR no DMO vs DM no DR	Microperimetry mesopic	19	0
DR no DMO vs DM no DR	Moorfields chart acuity	7	10
DR no DMO vs DM no DR	Near visual acuity	10	8
DR no DMO vs DM no DR	Pelli-Robson contrast sensitivity	4	10
DR no DMO vs DM no DR	Reading index	14	21
DR no DMO vs DM no DR	Smith-Kettlewell low luminance near visual acuity	9	15
DR with DMO vs DR no DMO	Distance visual acuity	17	7
DR with DMO vs DR no DMO	Low luminance visual acuity	6	10
DR with DMO vs DR no DMO	Matrix perimetry	8	0
DR with DMO vs DR no DMO	Microperimetry mesopic	7	0
DR with DMO vs DR no DMO	Moorfields chart acuity	4	11
DR with DMO vs DR no DMO	Near visual acuity	12	10
DR with DMO vs DR no DMO	Pelli-Robson contrast sensitivity	10	10
DR with DMO vs DR no DMO	Reading index	6	22
DR with DMO vs DR no DMO	Smith-Kettlewell low luminance near visual acuity	14	11

DM, diabetes mellitus; DMO, diabetic macular oedema; DR, diabetic retinopathy.

Distinguishing eyes with DR no DMO versus DM no DR (task 2), 19 of the top 30 models contained mesopic microperimetry. Other high-ranking tests included reading index, near visual acuity and matrix perimetry. In the no perimetry dataset, reading index was in 21 of the top 30 models and Smith-Kettlewell low luminance near visual acuity was in 15.

Distinguishing eyes with DR with DMO versus DR no DMO (task 3), 17 of the top 30 models in the ‘all tests’ dataset contained DVA. Smith-Kettlewell low luminance near visual acuity and near visual acuity both ranked highly. In the no perimetry dataset, reading index featured in 22 of the top 30 models and Moorfields chart acuity featured in 11.

For all tasks and both datasets, the highest-ranking models combined three visual function tests (ESM, online supplemental tables 3 and 4). For all tasks and both datasets, most models in the top 30 were three-way combinations of visual function tests. In the ‘all tests’ dataset, there were 2 two-way combinations for task 1 and four each for tasks 2 and 3. A single one-way model was in the top 30 for task 3, containing matrix perimetry. In the no perimetry dataset, there were seven two-way models for each task. One-way models containing reading index were in the top 30 for tasks 1 and task 3.

## Discussion

### Main findings

We developed ensemble ML models that combine measurements from up to three visual function tests with age and sex. Models distinguished between stages of diabetic eye disease with performance similar to that obtained using all nine visual function tests.^7^ Our listings of model performance will assist researchers in selecting the most appropriate test combinations for the task. They will also help in selecting alternatives if particular tests are too expensive or time-consuming, are unpopular with participants or are less effective among particular groups (eg, those with reduced cognitive function).

We identified DVA, Pelli-Robson contrast sensitivity and low luminance visual acuity as the most highly ranked tests for distinguishing DM no DR from no DM. For distinguishing DR no DMO from DM no DR mesopic microperimetry, near visual acuity, matrix microperimetry and reading index were highly ranked. For distinguishing DR with DMO from DR no DMO, DVA was highly ranked. Smith-Kettlewell low luminance near visual acuity and near visual acuity ranked highly. In the absence of perimetry, reading index ranked highly for all three tasks.

We have described in detail the distribution of visual function measurements conducted on over 1000 individuals, across a spectrum ranging from no diabetes to severe diabetic eye disease. These statistics constitute a comprehensive reference for those planning clinical trials or diagnostic test accuracy studies who are planning to use visual function as an outcome.

### Placing the findings in context

While DVA remains the most common functional endpoint in DR studies, it is well recognised that this measure alone fails to capture the full spectrum of functional deficits experienced by patients.^5^ Previous studies have demonstrated that other visual function measures—including contrast sensitivity, reading speed and microperimetry—may detect impairments earlier or provide better correlation with patient-reported outcomes and quality of life.^16^,^18^ However, how combinations of visual function tests relate to specific DR stages has not been addressed to our knowledge. Our application of ensemble ML approaches to identify optimal combinations of up to three visual function measures provides a novel contribution, aligned with growing interest in personalised, multimodal assessments of DR.^19^

Our finding that models containing DVA, contrast sensitivity, microperimetry and reading index achieved excellent discrimination (AUC ≥0.94 across tasks) is consistent with earlier reports showing the added value of these tests.^20^ Reading index, a derivative of reading speed, is highlighted as being useful for most of the tasks. It is a complex visual function that depends on multiple visual and cognitive processes, including high-resolution central vision, contrast sensitivity, eye movement control and visual information processing.^21 22^ As DR progresses, structural and functional changes in the retina disrupt these processes, leading to measurable declines in reading performance. DR-related macular changes also directly impair foveal function, which is critical for resolving fine detail required for reading. Fluid accumulation, intraretinal cysts and hard exudates distort the photoreceptor layer and retinal architecture, degrading spatial resolution and contrast detection at the fovea. As a result, patients tend to require longer fixation times to recognise letters and words, reducing reading speed. DR is also associated with diffuse neurodegenerative changes early in the disease course, including ganglion cell loss and disruption of inner retinal synapses, even in the absence of visible vascular lesions.^23^,^25^ These neurovascular changes impair signal transmission, leading to slower processing of visual information. DR may also affect ocular motor control, increasing the frequency of fixation instability, saccadic intrusions and regressions during reading.^21^ These inefficiencies in eye movements further slow reading, as the visual system struggles to maintain a stable image of text on the retina. Reduced contrast sensitivity, which our study also highlights as important, impairs the rapid decoding of text required for fluent reading.

Apart from DVA, contrast sensitivity and low luminance near visual acuity (SKILL card) are highlighted as important in both task 1 and task 3, possibly reflecting the susceptibility of the inner retinal layers to metabolic and inflammatory disturbances in diabetes. Chronic hyperglycaemia triggers oxidative stress, low-grade inflammation and accumulation of advanced glycation end products, leading to neural apoptosis and microvascular dysfunction.^23 26^ These changes disrupt retinal signal transmission, particularly affecting pathways responsible for detecting low contrast stimuli.

In practical terms, our model rankings facilitate selection of test combinations suitable for busy clinical environments. It is possible to conduct two or three chart-based tests in the time taken to perform a single perimetry test, so these present useful options under time pressure. To set our study in a broader context, our top-ranking models for tasks 2 and 3, with AUC ≥0.99 achieved comparable performance to imaging-based models to detect DR^27 28^ and DMO.^29^ Notably, meta-analysis of performance of an FDA-approved AI system for detecting DR indicates it achieves AUC of 0.95.^28^

### Strengths, limitations and further work

The study dataset was large and covered a wide range of visual function tests, enabling a thorough exploration of the distribution of measures across groups. This included a larger and more diverse normal (no DM) group than in many studies. Quantifying the variation in normal eyes is key to determining effect and sample size estimation for future studies.

Our ensemble modelling approach produced substantially better performance than more standard logistic regression approaches, indicating that more flexible models are required to accurately distinguish diabetic eye disease status.

Although crossvalidation is inherent to the SuperLearner algorithm, a true validation of model performance requires external datasets. Other datasets are unlikely to contain all nine of the visual function tests used here and detailed functional studies tend to be small. However, it may be possible to rank models by triangulation, validating one-way, two-way or three-way combinations of tests for each task using data from individual studies, and then constructing a composite ranking across multiple datasets, akin to meta-analysis. This approach could be extended to include newly developed visual function tests not available for our study but subsequently associated with DR status, for example, quantitative contrast sensitivity function or flicker electroretinogram tests.^30 31^

This study was cross-sectional, but it would be valuable to apply these approaches to longitudinal data to determine whether visual function measurements can be used to predict morphological changes as diabetic eye disease progresses.

## Conclusion

Using a dataset comprising over 1000 individuals, we demonstrated ML models to classify status of diabetic eye disease with high performance, using just age, sex and at most three from a battery of nine visual function tests. These had similar performance to models using the entire battery. We also provide detailed descriptions of the distribution of each visual function measure for use as a reference when designing studies with visual function as the outcome.

## Supplementary material

10.1136/bmjophth-2025-002536 online supplemental file 1

## Data Availability

Data are available upon reasonable request.
