# Supplementary material for: Selecting measures of visual function to classify diabetic retinopathy status: a cross-sectional study
Source: BMJ Open Ophthalmol. 2026 Jan 20;11(1):e002536. doi: 10.1136/bmjophth-2025-002536 (PMC12820872; doi:10.1136/bmjophth-2025-002536)
Supplement: online supplemental file 1 [file bmjophth-11-1-s001.docx]

Selecting measures of visual function to classify diabetic retinopathy status: a cross-sectional study

David M. Wright^1^, Usha Chakravarthy^1^, Radha Das^1^, Katie W. Graham^1^, Timos T. Naskas^1^, Tunde Peto^1^, Ruth E. Hogg^1^

^1^Centre for Public Health, Queen’s University Belfast

# Electronic Supplementary Material

## Supplementary methods – visual function testing

### Distance visual acuity

Monocular DVA was evaluated using Early Treatment for Diabetic Retinopathy Study (EDTRS)charts in a light box (Precision Vision, USA) at 4 m.

### Near visual acuity

NVA measured monocularly using Bailey–Lovie near word reading charts at 25 cm with the appropriate room lights on

### Reading Index

The reading speed was assessed separately in each eye using 2 sets of modified Bailey-Lovie reading speed charts presented as transparencies with black text and corresponding reading speed score sheets^11^. The reading speed assessment was tested in subjects using the appropriate reading addition worn over the protocol refraction at 4 meters providing the BCVA. The reading speed chart selected for the reading speed assessment should exhibit text of a print size two logarithmic steps larger than the subjects measured near word visual acuity in the tested eye. The reading index is the reading speed divided by the size of print read and thus makes allowance for the visual angle^10^.

### Distance low-luminance visual acuity

This was measured at 4 m using the EDTRS chart and a 2.0 log neutral-density trial lens was inserted over the final distance refraction result.

### Near low‑luminance visual acuity

This was measured at 25cm and the Smith-Kettlewell Institute low-luminance (SKILL) card was used^12^. The number of letters read in each card was recorded, which was held approximately 40 cm from the patient’s eye wearing the appropriate reading addition with high contrast letters facing the patient under normal room lighting. The number of letters were recorded correctly identified, assuming all above the first line read perfectly (5 out of 5 correct) are also perfect.

### Contrast sensitivity

This was measured monocularly using Pelli–Robson charts (Clement Clarke International, UK) viewed at 1 m.

### Moorfields Acuity chart

Described in ^13^.

### Frequency doubling technology perimetry

The central visual field was assessed using a frequency doubling technology (FDT) Matrix perimeter (Carl Zeiss Meditec, USA) using the 24–2 threshold test.

### Microperimetry Macular Integrity Assessment

Performed using a MAIA macular integrity assessment system (CenterVue, Italy). A red circular fixation target of 18 diameter was used and Goldman III stimuli were presented against a background of 1.27 cd/m2 using a 4-2-threshold strategy. We used a customized stimulus grid designed specifically for the assessment of the macular region in age related macular degeneration. It consisted of 45 points, designed in a manner to allow a relatively regular sampling density throughout the region, but with a slightly increased density towards the fovea^14^.

## Supplementary Tables

ESM Table 1 Distribution of visual function measurements by diabetes and retinopathy status. Eyes that received all nine visual function tests.

| Description | Diabetes/retinopathy status | N | Mean | SD | 2.5% quantile | 25% quantile | 50% quantile | 75% quantile | 97.5% quantile |
| --- | --- | --- | --- | --- | --- | --- | --- | --- | --- |
| Best-corrected visual acuity at 4m | No DM | 324 | 88.21 | 4.83 | 79.00 | 85.00 | 89.00 | 92.00 | 95.00 |
| Best-corrected visual acuity at 4m | DM no DR | 116 | 83.72 | 7.68 | 67.38 | 81.00 | 85.00 | 88.00 | 93.00 |
| Best-corrected visual acuity at 4m | DR no DMO | 91 | 83.25 | 7.85 | 66.25 | 80.00 | 85.00 | 90.00 | 94.00 |
| Best-corrected visual acuity at 4m | DR with DMO | 52 | 74.19 | 12.26 | 46.10 | 66.75 | 77.00 | 84.00 | 88.72 |
| Low luminance visual acuity at 4m | No DM | 322 | 76.01 | 5.42 | 65.00 | 73.00 | 77.00 | 80.00 | 85.00 |
| Low luminance visual acuity at 4m | DM no DR | 116 | 70.43 | 9.75 | 52.88 | 66.75 | 72.00 | 75.00 | 82.12 |
| Low luminance visual acuity at 4m | DR no DMO | 91 | 69.60 | 8.31 | 49.00 | 65.50 | 70.00 | 75.00 | 82.25 |
| Low luminance visual acuity at 4m | DR with DMO | 52 | 58.42 | 17.41 | 2.28 | 54.75 | 64.00 | 70.00 | 75.72 |
| Moorfields chart acuity at 4m | No DM | 181 | 37.82 | 5.11 | 26.50 | 35.00 | 39.00 | 42.00 | 45.00 |
| Moorfields chart acuity at 4m | DM no DR | 74 | 32.93 | 7.33 | 18.48 | 29.25 | 35.00 | 39.00 | 44.00 |
| Moorfields chart acuity at 4m | DR no DMO | 68 | 33.00 | 7.74 | 19.00 | 29.00 | 34.00 | 39.00 | 45.00 |
| Moorfields chart acuity at 4m | DR with DMO | 33 | 23.67 | 9.83 | 3.20 | 18.00 | 25.00 | 30.00 | 37.40 |
| Pelli-Robson contrast sensitivity | No DM | 324 | 1.65 | 0.14 | 1.35 | 1.65 | 1.65 | 1.80 | 1.80 |
| Pelli-Robson contrast sensitivity | DM no DR | 115 | 1.50 | 0.19 | 1.05 | 1.35 | 1.50 | 1.65 | 1.67 |
| Pelli-Robson contrast sensitivity | DR no DMO | 91 | 1.48 | 0.14 | 1.20 | 1.35 | 1.50 | 1.65 | 1.65 |
| Pelli-Robson contrast sensitivity | DR with DMO | 51 | 1.37 | 0.19 | 1.05 | 1.27 | 1.35 | 1.50 | 1.65 |
| Near visual acuity | No DM | 324 | 0.10 | 0.09 | 0.00 | 0.00 | 0.10 | 0.10 | 0.30 |
| Near visual acuity | DM no DR | 116 | 0.19 | 0.17 | 0.00 | 0.10 | 0.20 | 0.20 | 0.70 |
| Near visual acuity | DR no DMO | 91 | 0.22 | 0.15 | 0.02 | 0.10 | 0.20 | 0.30 | 0.60 |
| Near visual acuity | DR with DMO | 52 | 0.41 | 0.26 | 0.03 | 0.27 | 0.30 | 0.60 | 0.97 |
| Smith-Kettlewell low luminance near visual acuity | No DM | 324 | 29.76 | 5.57 | 20.00 | 26.00 | 29.00 | 33.00 | 41.00 |
| Smith-Kettlewell low luminance near visual acuity | DM no DR | 115 | 34.89 | 8.26 | 22.00 | 29.00 | 34.00 | 40.00 | 56.15 |
| Smith-Kettlewell low luminance near visual acuity | DR no DMO | 91 | 36.22 | 9.14 | 23.00 | 29.00 | 35.00 | 42.50 | 53.00 |
| Smith-Kettlewell low luminance near visual acuity | DR with DMO | 52 | 37.98 | 12.90 | 17.27 | 30.00 | 35.00 | 45.25 | 61.63 |
| Reading index | No DM | 322 | 47.19 | 8.92 | 30.66 | 41.85 | 46.84 | 52.71 | 66.74 |
| Reading index | DM no DR | 112 | 39.44 | 9.91 | 21.51 | 32.24 | 40.03 | 46.20 | 56.03 |
| Reading index | DR no DMO | 83 | 37.85 | 11.32 | 15.64 | 30.02 | 39.54 | 46.61 | 55.11 |
| Reading index | DR with DMO | 51 | 32.28 | 13.13 | 9.82 | 22.66 | 32.97 | 41.65 | 57.38 |
| Matrix perimetry mean deviation | No DM | 324 | -1.90 | 3.18 | -8.64 | -3.56 | -1.51 | 0.15 | 3.73 |
| Matrix perimetry mean deviation | DM no DR | 116 | -3.87 | 4.61 | -16.75 | -5.72 | -3.16 | -1.01 | 2.50 |
| Matrix perimetry mean deviation | DR no DMO | 91 | -4.00 | 4.18 | -14.81 | -5.94 | -3.95 | -0.75 | 2.30 |
| Matrix perimetry mean deviation | DR with DMO | 52 | -4.61 | 3.96 | -14.79 | -6.67 | -4.09 | -2.13 | 2.03 |
| Matrix perimetry pattern standard deviation | No DM | 324 | 3.24 | 0.96 | 2.09 | 2.65 | 3.01 | 3.67 | 5.96 |
| Matrix perimetry pattern standard deviation | DM no DR | 116 | 4.04 | 1.93 | 2.26 | 2.91 | 3.60 | 4.40 | 10.39 |
| Matrix perimetry pattern standard deviation | DR no DMO | 91 | 4.19 | 1.50 | 2.56 | 3.16 | 3.72 | 4.95 | 7.47 |
| Matrix perimetry pattern standard deviation | DR with DMO | 52 | 4.52 | 1.57 | 2.55 | 3.48 | 4.08 | 5.24 | 7.99 |
| Microperimetry average sensitivity | No DM | 302 | 27.13 | 1.60 | 23.61 | 26.10 | 27.20 | 28.30 | 29.89 |
| Microperimetry average sensitivity | DM no DR | 104 | 25.87 | 2.31 | 20.40 | 24.60 | 26.00 | 27.70 | 29.00 |
| Microperimetry average sensitivity | DR no DMO | 86 | 25.37 | 2.84 | 17.80 | 23.92 | 25.95 | 27.25 | 29.06 |
| Microperimetry average sensitivity | DR with DMO | 50 | 22.81 | 3.18 | 15.56 | 20.78 | 23.15 | 24.78 | 27.26 |
| Microperimetry fixation area 95% | No DM | 322 | 3.61 | 4.82 | 0.30 | 0.90 | 1.90 | 4.00 | 17.27 |
| Microperimetry fixation area 95% | DM no DR | 111 | 5.14 | 8.26 | 0.40 | 1.05 | 2.10 | 5.20 | 22.60 |
| Microperimetry fixation area 95% | DR no DMO | 89 | 7.08 | 10.86 | 0.30 | 1.20 | 3.40 | 6.80 | 32.80 |
| Microperimetry fixation area 95% | DR with DMO | 52 | 8.10 | 8.88 | 0.46 | 1.67 | 4.25 | 11.07 | 29.75 |
| Microperimetry central 5 points mean sensitivity | No DM | 322 | 27.71 | 1.79 | 24.20 | 26.60 | 27.80 | 29.00 | 30.80 |
| Microperimetry central 5 points mean sensitivity | DM no DR | 111 | 26.21 | 2.62 | 19.95 | 24.80 | 26.60 | 28.10 | 30.05 |
| Microperimetry central 5 points mean sensitivity | DR no DMO | 89 | 25.63 | 3.43 | 18.48 | 24.00 | 26.20 | 28.20 | 29.88 |
| Microperimetry central 5 points mean sensitivity | DR with DMO | 52 | 20.71 | 5.46 | 5.85 | 18.35 | 22.00 | 24.50 | 27.55 |

ESM Table 2. Distribution of visual function measurements by diabetes and retinopathy status. Eyes that did not receive perimetry.

| Description | Diabetes/retinopathy status | N | Mean | SD | 2.5% quantile | 25% quantile | 50% quantile | 75% quantile | 97.5% quantile |
| --- | --- | --- | --- | --- | --- | --- | --- | --- | --- |
| Best-corrected visual acuity at 4m | No DM | 1317 | 85.73 | 6.71 | 72.00 | 83.00 | 87.00 | 90.00 | 95.00 |
| Best-corrected visual acuity at 4m | DM no DR | 278 | 82.64 | 11.63 | 61.92 | 80.00 | 85.00 | 88.00 | 94.00 |
| Best-corrected visual acuity at 4m | DR no DMO | 216 | 83.53 | 7.35 | 67.38 | 80.00 | 85.00 | 89.00 | 94.00 |
| Best-corrected visual acuity at 4m | DR with DMO | 90 | 73.91 | 12.37 | 44.22 | 67.25 | 77.00 | 83.75 | 90.00 |
| Low luminance visual acuity at 4m | No DM | 1315 | 72.91 | 7.63 | 55.00 | 69.00 | 74.00 | 78.00 | 84.00 |
| Low luminance visual acuity at 4m | DM no DR | 278 | 69.79 | 11.75 | 43.00 | 67.00 | 72.00 | 75.00 | 83.00 |
| Low luminance visual acuity at 4m | DR no DMO | 216 | 70.25 | 8.12 | 49.00 | 66.75 | 70.00 | 75.00 | 83.00 |
| Low luminance visual acuity at 4m | DR with DMO | 90 | 59.07 | 16.38 | 3.00 | 51.00 | 63.50 | 70.00 | 76.00 |
| Moorfields chart acuity at 4m | No DM | 1019 | 35.68 | 6.81 | 19.00 | 32.00 | 36.00 | 40.00 | 45.00 |
| Moorfields chart acuity at 4m | DM no DR | 192 | 33.13 | 8.34 | 13.88 | 30.00 | 35.00 | 39.00 | 45.00 |
| Moorfields chart acuity at 4m | DR no DMO | 157 | 34.04 | 7.12 | 19.90 | 30.00 | 35.00 | 40.00 | 45.00 |
| Moorfields chart acuity at 4m | DR with DMO | 57 | 22.82 | 11.28 | 0.40 | 15.00 | 25.00 | 30.00 | 40.20 |
| Pelli-Robson contrast sensitivity | No DM | 1315 | 1.56 | 0.17 | 1.20 | 1.50 | 1.65 | 1.65 | 1.80 |
| Pelli-Robson contrast sensitivity | DM no DR | 274 | 1.51 | 0.21 | 1.05 | 1.35 | 1.50 | 1.65 | 1.80 |
| Pelli-Robson contrast sensitivity | DR no DMO | 216 | 1.48 | 0.14 | 1.20 | 1.35 | 1.50 | 1.65 | 1.65 |
| Pelli-Robson contrast sensitivity | DR with DMO | 88 | 1.35 | 0.23 | 0.78 | 1.20 | 1.35 | 1.50 | 1.65 |
| Near visual acuity | No DM | 1316 | 0.16 | 0.13 | 0.00 | 0.10 | 0.10 | 0.20 | 0.50 |
| Near visual acuity | DM no DR | 276 | 0.21 | 0.19 | 0.00 | 0.10 | 0.20 | 0.30 | 0.70 |
| Near visual acuity | DR no DMO | 216 | 0.20 | 0.14 | 0.00 | 0.10 | 0.20 | 0.30 | 0.50 |
| Near visual acuity | DR with DMO | 90 | 0.41 | 0.27 | 0.00 | 0.20 | 0.30 | 0.60 | 1.08 |
| Smith-Kettlewell low luminance near visual acuity | No DM | 1315 | 32.31 | 7.63 | 20.00 | 27.00 | 31.00 | 36.00 | 50.15 |
| Smith-Kettlewell low luminance near visual acuity | DM no DR | 277 | 33.36 | 8.67 | 19.00 | 28.00 | 33.00 | 37.00 | 55.00 |
| Smith-Kettlewell low luminance near visual acuity | DR no DMO | 216 | 35.18 | 8.77 | 20.75 | 29.00 | 35.00 | 40.00 | 54.25 |
| Smith-Kettlewell low luminance near visual acuity | DR with DMO | 90 | 39.01 | 12.24 | 18.45 | 30.00 | 37.50 | 49.00 | 61.32 |
| Reading index | No DM | 1306 | 45.06 | 9.53 | 26.69 | 39.15 | 45.00 | 51.06 | 62.77 |
| Reading index | DM no DR | 253 | 39.95 | 10.24 | 16.31 | 32.64 | 40.93 | 47.22 | 58.35 |
| Reading index | DR no DMO | 187 | 37.62 | 10.88 | 15.61 | 29.31 | 38.35 | 44.84 | 55.11 |
| Reading index | DR with DMO | 88 | 32.78 | 13.67 | 9.76 | 21.84 | 32.42 | 44.76 | 59.22 |

ESM Table 3. Performance of combinations of visual function tests by task. All tests.

| Sample | Task | Var1 | Var2 | Var3 | Rank | N | AUC | AUCLower | AUCUpper | GLMAUC |
| --- | --- | --- | --- | --- | --- | --- | --- | --- | --- | --- |
| All tests | DM no DR vs. No DM | Distance visual acuity | Low luminance visual acuity | Microperimetry mesopic | 8 | 404 | 0.98 | 0.98 | 0.99 | 0.72 |
| All tests | DM no DR vs. No DM | Distance visual acuity | Low luminance visual acuity | Moorfields chart acuity | 28 | 253 | 0.96 | 0.94 | 0.98 | 0.71 |
| All tests | DM no DR vs. No DM | Distance visual acuity | Low luminance visual acuity | Near visual acuity | 5 | 438 | 0.99 | 0.98 | 1.00 | 0.73 |
| All tests | DM no DR vs. No DM | Distance visual acuity | Low luminance visual acuity | Pelli-Robson contrast sensitivity | 1 | 437 | 1.00 | 0.99 | 1.00 | 0.77 |
| All tests | DM no DR vs. No DM | Distance visual acuity | Low luminance visual acuity | Reading index | 2 | 432 | 0.99 | 0.99 | 1.00 | 0.76 |
| All tests | DM no DR vs. No DM | Distance visual acuity | Low luminance visual acuity | Smith-Kettlewell low luminance near visual acuity | 4 | 437 | 0.99 | 0.98 | 1.00 | 0.75 |
| All tests | DM no DR vs. No DM | Distance visual acuity | Low luminance visual acuity | NA | 12 | 438 | 0.98 | 0.97 | 0.99 | 0.72 |
| All tests | DM no DR vs. No DM | Distance visual acuity | Matrix perimetry | Near visual acuity | 30 | 440 | 0.96 | 0.94 | 0.98 | 0.74 |
| All tests | DM no DR vs. No DM | Distance visual acuity | Microperimetry mesopic | Moorfields chart acuity | 21 | 238 | 0.97 | 0.95 | 0.99 | 0.68 |
| All tests | DM no DR vs. No DM | Distance visual acuity | Microperimetry mesopic | Pelli-Robson contrast sensitivity | 14 | 405 | 0.98 | 0.97 | 0.99 | 0.77 |
| All tests | DM no DR vs. No DM | Distance visual acuity | Moorfields chart acuity | Near visual acuity | 17 | 255 | 0.97 | 0.96 | 0.99 | 0.72 |
| All tests | DM no DR vs. No DM | Distance visual acuity | Near visual acuity | Pelli-Robson contrast sensitivity | 27 | 439 | 0.96 | 0.94 | 0.98 | 0.77 |
| All tests | DM no DR vs. No DM | Distance visual acuity | Near visual acuity | Reading index | 33 | 434 | 0.96 | 0.94 | 0.97 | 0.76 |
| All tests | DM no DR vs. No DM | Distance visual acuity | Near visual acuity | Smith-Kettlewell low luminance near visual acuity | 15 | 439 | 0.98 | 0.97 | 0.99 | 0.75 |
| All tests | DM no DR vs. No DM | Distance visual acuity | Pelli-Robson contrast sensitivity | Reading index | 18 | 433 | 0.97 | 0.96 | 0.99 | 0.80 |
| All tests | DM no DR vs. No DM | Distance visual acuity | Pelli-Robson contrast sensitivity | Smith-Kettlewell low luminance near visual acuity | 11 | 438 | 0.98 | 0.97 | 0.99 | 0.77 |
| All tests | DM no DR vs. No DM | Distance visual acuity | Reading index | Smith-Kettlewell low luminance near visual acuity | 9 | 433 | 0.98 | 0.98 | 0.99 | 0.78 |
| All tests | DM no DR vs. No DM | Distance visual acuity | Smith-Kettlewell low luminance near visual acuity | NA | 19 | 439 | 0.97 | 0.96 | 0.98 | 0.75 |
| All tests | DM no DR vs. No DM | Low luminance visual acuity | Microperimetry mesopic | Moorfields chart acuity | 13 | 236 | 0.98 | 0.97 | 0.99 | 0.68 |
| All tests | DM no DR vs. No DM | Low luminance visual acuity | Microperimetry mesopic | Near visual acuity | 24 | 404 | 0.97 | 0.95 | 0.98 | 0.72 |
| All tests | DM no DR vs. No DM | Low luminance visual acuity | Microperimetry mesopic | Pelli-Robson contrast sensitivity | 32 | 403 | 0.96 | 0.94 | 0.98 | 0.75 |
| All tests | DM no DR vs. No DM | Low luminance visual acuity | Moorfields chart acuity | Pelli-Robson contrast sensitivity | 22 | 253 | 0.97 | 0.95 | 0.99 | 0.72 |
| All tests | DM no DR vs. No DM | Low luminance visual acuity | Near visual acuity | Pelli-Robson contrast sensitivity | 20 | 437 | 0.97 | 0.96 | 0.98 | 0.76 |
| All tests | DM no DR vs. No DM | Low luminance visual acuity | Pelli-Robson contrast sensitivity | Reading index | 16 | 431 | 0.97 | 0.96 | 0.99 | 0.79 |
| All tests | DM no DR vs. No DM | Low luminance visual acuity | Pelli-Robson contrast sensitivity | Smith-Kettlewell low luminance near visual acuity | 7 | 436 | 0.99 | 0.98 | 0.99 | 0.77 |
| All tests | DM no DR vs. No DM | Matrix perimetry | Microperimetry mesopic | Pelli-Robson contrast sensitivity | 35 | 405 | 0.95 | 0.94 | 0.97 | 0.76 |
| All tests | DM no DR vs. No DM | Microperimetry mesopic | Moorfields chart acuity | Near visual acuity | 26 | 238 | 0.96 | 0.94 | 0.98 | 0.70 |
| All tests | DM no DR vs. No DM | Microperimetry mesopic | Moorfields chart acuity | Pelli-Robson contrast sensitivity | 23 | 238 | 0.97 | 0.95 | 0.99 | 0.70 |
| All tests | DM no DR vs. No DM | Microperimetry mesopic | Moorfields chart acuity | Smith-Kettlewell low luminance near visual acuity | 10 | 238 | 0.98 | 0.97 | 1.00 | 0.70 |
| All tests | DM no DR vs. No DM | Microperimetry mesopic | Near visual acuity | Pelli-Robson contrast sensitivity | 3 | 405 | 0.99 | 0.99 | 1.00 | 0.75 |
| All tests | DM no DR vs. No DM | Microperimetry mesopic | Pelli-Robson contrast sensitivity | Reading index | 25 | 400 | 0.96 | 0.95 | 0.98 | 0.78 |
| All tests | DM no DR vs. No DM | Microperimetry mesopic | Pelli-Robson contrast sensitivity | Smith-Kettlewell low luminance near visual acuity | 6 | 405 | 0.99 | 0.98 | 1.00 | 0.75 |
| All tests | DM no DR vs. No DM | Microperimetry mesopic | Pelli-Robson contrast sensitivity | NA | 34 | 405 | 0.96 | 0.94 | 0.97 | 0.75 |
| All tests | DM no DR vs. No DM | Moorfields chart acuity | Near visual acuity | Smith-Kettlewell low luminance near visual acuity | 29 | 254 | 0.96 | 0.94 | 0.98 | 0.74 |
| All tests | DM no DR vs. No DM | Near visual acuity | Pelli-Robson contrast sensitivity | Smith-Kettlewell low luminance near visual acuity | 31 | 438 | 0.96 | 0.94 | 0.98 | 0.77 |
| All tests | DR no DMO vs. DM no DR | Distance visual acuity | Low luminance visual acuity | Matrix perimetry | 37 | 207 | 1.00 | 1.00 | 1.00 | 0.60 |
| All tests | DR no DMO vs. DM no DR | Distance visual acuity | Low luminance visual acuity | Microperimetry mesopic | 26 | 190 | 1.00 | 1.00 | 1.00 | 0.64 |
| All tests | DR no DMO vs. DM no DR | Distance visual acuity | Low luminance visual acuity | Near visual acuity | 48 | 207 | 1.00 | 0.99 | 1.00 | 0.58 |
| All tests | DR no DMO vs. DM no DR | Distance visual acuity | Low luminance visual acuity | Smith-Kettlewell low luminance near visual acuity | 64 | 206 | 0.99 | 0.98 | 1.00 | 0.60 |
| All tests | DR no DMO vs. DM no DR | Distance visual acuity | Matrix perimetry | Microperimetry mesopic | 26 | 190 | 1.00 | 1.00 | 1.00 | 0.63 |
| All tests | DR no DMO vs. DM no DR | Distance visual acuity | Matrix perimetry | Near visual acuity | 24 | 207 | 1.00 | 1.00 | 1.00 | 0.60 |
| All tests | DR no DMO vs. DM no DR | Distance visual acuity | Matrix perimetry | Pelli-Robson contrast sensitivity | 70 | 206 | 0.99 | 0.97 | 1.00 | 0.61 |
| All tests | DR no DMO vs. DM no DR | Distance visual acuity | Matrix perimetry | Reading index | 32 | 195 | 1.00 | 1.00 | 1.00 | 0.58 |
| All tests | DR no DMO vs. DM no DR | Distance visual acuity | Matrix perimetry | Smith-Kettlewell low luminance near visual acuity | 33 | 206 | 1.00 | 1.00 | 1.00 | 0.60 |
| All tests | DR no DMO vs. DM no DR | Distance visual acuity | Matrix perimetry | NA | 49 | 207 | 1.00 | 0.99 | 1.00 | 0.59 |
| All tests | DR no DMO vs. DM no DR | Distance visual acuity | Microperimetry mesopic | Moorfields chart acuity | 80 | 130 | 0.97 | 0.95 | 0.99 | 0.65 |
| All tests | DR no DMO vs. DM no DR | Distance visual acuity | Microperimetry mesopic | Near visual acuity | 1 | 190 | 1.00 | 1.00 | 1.00 | 0.64 |
| All tests | DR no DMO vs. DM no DR | Distance visual acuity | Microperimetry mesopic | Pelli-Robson contrast sensitivity | 40 | 189 | 1.00 | 1.00 | 1.00 | 0.63 |
| All tests | DR no DMO vs. DM no DR | Distance visual acuity | Microperimetry mesopic | Reading index | 1 | 179 | 1.00 | 1.00 | 1.00 | 0.64 |
| All tests | DR no DMO vs. DM no DR | Distance visual acuity | Microperimetry mesopic | Smith-Kettlewell low luminance near visual acuity | 1 | 190 | 1.00 | 1.00 | 1.00 | 0.63 |
| All tests | DR no DMO vs. DM no DR | Distance visual acuity | Microperimetry mesopic | NA | 1 | 190 | 1.00 | 1.00 | 1.00 | 0.63 |
| All tests | DR no DMO vs. DM no DR | Distance visual acuity | Moorfields chart acuity | Smith-Kettlewell low luminance near visual acuity | 36 | 141 | 1.00 | 1.00 | 1.00 | 0.63 |
| All tests | DR no DMO vs. DM no DR | Distance visual acuity | Near visual acuity | Pelli-Robson contrast sensitivity | 72 | 206 | 0.98 | 0.97 | 1.00 | 0.59 |
| All tests | DR no DMO vs. DM no DR | Distance visual acuity | Near visual acuity | Reading index | 81 | 195 | 0.97 | 0.95 | 0.99 | 0.59 |
| All tests | DR no DMO vs. DM no DR | Distance visual acuity | Near visual acuity | Smith-Kettlewell low luminance near visual acuity | 42 | 206 | 1.00 | 1.00 | 1.00 | 0.60 |
| All tests | DR no DMO vs. DM no DR | Distance visual acuity | Pelli-Robson contrast sensitivity | Reading index | 83 | 194 | 0.96 | 0.94 | 0.99 | 0.58 |
| All tests | DR no DMO vs. DM no DR | Distance visual acuity | Pelli-Robson contrast sensitivity | Smith-Kettlewell low luminance near visual acuity | 54 | 205 | 0.99 | 0.99 | 1.00 | 0.60 |
| All tests | DR no DMO vs. DM no DR | Distance visual acuity | Reading index | Smith-Kettlewell low luminance near visual acuity | 79 | 194 | 0.97 | 0.95 | 0.99 | 0.58 |
| All tests | DR no DMO vs. DM no DR | Distance visual acuity | Smith-Kettlewell low luminance near visual acuity | NA | 52 | 206 | 0.99 | 0.99 | 1.00 | 0.59 |
| All tests | DR no DMO vs. DM no DR | Low luminance visual acuity | Matrix perimetry | Moorfields chart acuity | 89 | 142 | 0.95 | 0.92 | 0.99 | 0.63 |
| All tests | DR no DMO vs. DM no DR | Low luminance visual acuity | Matrix perimetry | Near visual acuity | 55 | 207 | 0.99 | 0.99 | 1.00 | 0.60 |
| All tests | DR no DMO vs. DM no DR | Low luminance visual acuity | Matrix perimetry | Pelli-Robson contrast sensitivity | 73 | 206 | 0.98 | 0.96 | 1.00 | 0.61 |
| All tests | DR no DMO vs. DM no DR | Low luminance visual acuity | Matrix perimetry | Reading index | 1 | 195 | 1.00 | 1.00 | 1.00 | 0.57 |
| All tests | DR no DMO vs. DM no DR | Low luminance visual acuity | Matrix perimetry | Smith-Kettlewell low luminance near visual acuity | 68 | 206 | 0.99 | 0.97 | 1.00 | 0.60 |
| All tests | DR no DMO vs. DM no DR | Low luminance visual acuity | Microperimetry mesopic | Moorfields chart acuity | 78 | 130 | 0.97 | 0.95 | 0.99 | 0.66 |
| All tests | DR no DMO vs. DM no DR | Low luminance visual acuity | Microperimetry mesopic | Near visual acuity | 1 | 190 | 1.00 | 1.00 | 1.00 | 0.64 |
| All tests | DR no DMO vs. DM no DR | Low luminance visual acuity | Microperimetry mesopic | Pelli-Robson contrast sensitivity | 57 | 189 | 0.99 | 0.98 | 1.00 | 0.64 |
| All tests | DR no DMO vs. DM no DR | Low luminance visual acuity | Microperimetry mesopic | Reading index | 1 | 179 | 1.00 | 1.00 | 1.00 | 0.64 |
| All tests | DR no DMO vs. DM no DR | Low luminance visual acuity | Microperimetry mesopic | Smith-Kettlewell low luminance near visual acuity | 35 | 190 | 1.00 | 1.00 | 1.00 | 0.63 |
| All tests | DR no DMO vs. DM no DR | Low luminance visual acuity | Microperimetry mesopic | NA | 26 | 190 | 1.00 | 1.00 | 1.00 | 0.63 |
| All tests | DR no DMO vs. DM no DR | Low luminance visual acuity | Moorfields chart acuity | Near visual acuity | 62 | 142 | 0.99 | 0.98 | 1.00 | 0.63 |
| All tests | DR no DMO vs. DM no DR | Low luminance visual acuity | Moorfields chart acuity | Reading index | 69 | 133 | 0.99 | 0.97 | 1.00 | 0.62 |
| All tests | DR no DMO vs. DM no DR | Low luminance visual acuity | Moorfields chart acuity | Smith-Kettlewell low luminance near visual acuity | 44 | 141 | 1.00 | 0.99 | 1.00 | 0.65 |
| All tests | DR no DMO vs. DM no DR | Low luminance visual acuity | Near visual acuity | Reading index | 1 | 195 | 1.00 | 1.00 | 1.00 | 0.58 |
| All tests | DR no DMO vs. DM no DR | Low luminance visual acuity | Pelli-Robson contrast sensitivity | Reading index | 87 | 194 | 0.96 | 0.93 | 0.98 | 0.57 |
| All tests | DR no DMO vs. DM no DR | Low luminance visual acuity | Pelli-Robson contrast sensitivity | Smith-Kettlewell low luminance near visual acuity | 86 | 205 | 0.96 | 0.93 | 0.98 | 0.60 |
| All tests | DR no DMO vs. DM no DR | Low luminance visual acuity | Reading index | Smith-Kettlewell low luminance near visual acuity | 38 | 194 | 1.00 | 1.00 | 1.00 | 0.58 |
| All tests | DR no DMO vs. DM no DR | Matrix perimetry | Microperimetry mesopic | Near visual acuity | 1 | 190 | 1.00 | 1.00 | 1.00 | 0.63 |
| All tests | DR no DMO vs. DM no DR | Matrix perimetry | Microperimetry mesopic | Reading index | 1 | 179 | 1.00 | 1.00 | 1.00 | 0.64 |
| All tests | DR no DMO vs. DM no DR | Matrix perimetry | Microperimetry mesopic | Smith-Kettlewell low luminance near visual acuity | 1 | 190 | 1.00 | 1.00 | 1.00 | 0.63 |
| All tests | DR no DMO vs. DM no DR | Matrix perimetry | Microperimetry mesopic | NA | 65 | 190 | 0.99 | 0.98 | 1.00 | 0.63 |
| All tests | DR no DMO vs. DM no DR | Matrix perimetry | Moorfields chart acuity | Near visual acuity | 51 | 142 | 0.99 | 0.99 | 1.00 | 0.64 |
| All tests | DR no DMO vs. DM no DR | Matrix perimetry | Moorfields chart acuity | Pelli-Robson contrast sensitivity | 53 | 142 | 0.99 | 0.99 | 1.00 | 0.63 |
| All tests | DR no DMO vs. DM no DR | Matrix perimetry | Moorfields chart acuity | Smith-Kettlewell low luminance near visual acuity | 1 | 141 | 1.00 | 1.00 | 1.00 | 0.64 |
| All tests | DR no DMO vs. DM no DR | Matrix perimetry | Moorfields chart acuity | NA | 59 | 142 | 0.99 | 0.98 | 1.00 | 0.63 |
| All tests | DR no DMO vs. DM no DR | Matrix perimetry | Near visual acuity | Pelli-Robson contrast sensitivity | 46 | 206 | 1.00 | 0.99 | 1.00 | 0.61 |
| All tests | DR no DMO vs. DM no DR | Matrix perimetry | Near visual acuity | Reading index | 45 | 195 | 1.00 | 0.99 | 1.00 | 0.58 |
| All tests | DR no DMO vs. DM no DR | Matrix perimetry | Near visual acuity | Smith-Kettlewell low luminance near visual acuity | 1 | 206 | 1.00 | 1.00 | 1.00 | 0.61 |
| All tests | DR no DMO vs. DM no DR | Matrix perimetry | Near visual acuity | NA | 74 | 207 | 0.98 | 0.96 | 1.00 | 0.60 |
| All tests | DR no DMO vs. DM no DR | Matrix perimetry | Pelli-Robson contrast sensitivity | Reading index | 1 | 194 | 1.00 | 1.00 | 1.00 | 0.58 |
| All tests | DR no DMO vs. DM no DR | Matrix perimetry | Pelli-Robson contrast sensitivity | Smith-Kettlewell low luminance near visual acuity | 56 | 205 | 0.99 | 0.99 | 1.00 | 0.61 |
| All tests | DR no DMO vs. DM no DR | Matrix perimetry | Pelli-Robson contrast sensitivity | NA | 76 | 206 | 0.97 | 0.96 | 0.99 | 0.61 |
| All tests | DR no DMO vs. DM no DR | Matrix perimetry | Reading index | Smith-Kettlewell low luminance near visual acuity | 1 | 194 | 1.00 | 1.00 | 1.00 | 0.58 |
| All tests | DR no DMO vs. DM no DR | Matrix perimetry | Reading index | NA | 63 | 195 | 0.99 | 0.98 | 1.00 | 0.58 |
| All tests | DR no DMO vs. DM no DR | Matrix perimetry | NA | NA | 50 | 207 | 1.00 | 0.99 | 1.00 | 0.60 |
| All tests | DR no DMO vs. DM no DR | Microperimetry mesopic | Moorfields chart acuity | Near visual acuity | 1 | 130 | 1.00 | 1.00 | 1.00 | 0.64 |
| All tests | DR no DMO vs. DM no DR | Microperimetry mesopic | Moorfields chart acuity | Pelli-Robson contrast sensitivity | 1 | 130 | 1.00 | 1.00 | 1.00 | 0.64 |
| All tests | DR no DMO vs. DM no DR | Microperimetry mesopic | Moorfields chart acuity | Reading index | 34 | 121 | 1.00 | 1.00 | 1.00 | 0.66 |
| All tests | DR no DMO vs. DM no DR | Microperimetry mesopic | Moorfields chart acuity | Smith-Kettlewell low luminance near visual acuity | 1 | 130 | 1.00 | 1.00 | 1.00 | 0.65 |
| All tests | DR no DMO vs. DM no DR | Microperimetry mesopic | Moorfields chart acuity | NA | 41 | 130 | 1.00 | 1.00 | 1.00 | 0.64 |
| All tests | DR no DMO vs. DM no DR | Microperimetry mesopic | Near visual acuity | Pelli-Robson contrast sensitivity | 1 | 189 | 1.00 | 1.00 | 1.00 | 0.64 |
| All tests | DR no DMO vs. DM no DR | Microperimetry mesopic | Near visual acuity | Reading index | 30 | 179 | 1.00 | 1.00 | 1.00 | 0.65 |
| All tests | DR no DMO vs. DM no DR | Microperimetry mesopic | Near visual acuity | Smith-Kettlewell low luminance near visual acuity | 66 | 190 | 0.99 | 0.97 | 1.00 | 0.63 |
| All tests | DR no DMO vs. DM no DR | Microperimetry mesopic | Near visual acuity | NA | 61 | 190 | 0.99 | 0.98 | 1.00 | 0.63 |
| All tests | DR no DMO vs. DM no DR | Microperimetry mesopic | Pelli-Robson contrast sensitivity | Reading index | 43 | 178 | 1.00 | 1.00 | 1.00 | 0.64 |
| All tests | DR no DMO vs. DM no DR | Microperimetry mesopic | Pelli-Robson contrast sensitivity | Smith-Kettlewell low luminance near visual acuity | 71 | 189 | 0.99 | 0.97 | 1.00 | 0.64 |
| All tests | DR no DMO vs. DM no DR | Microperimetry mesopic | Pelli-Robson contrast sensitivity | NA | 85 | 189 | 0.96 | 0.93 | 0.98 | 0.64 |
| All tests | DR no DMO vs. DM no DR | Microperimetry mesopic | Reading index | Smith-Kettlewell low luminance near visual acuity | 1 | 179 | 1.00 | 1.00 | 1.00 | 0.64 |
| All tests | DR no DMO vs. DM no DR | Microperimetry mesopic | Reading index | NA | 1 | 179 | 1.00 | 1.00 | 1.00 | 0.64 |
| All tests | DR no DMO vs. DM no DR | Microperimetry mesopic | Smith-Kettlewell low luminance near visual acuity | NA | 77 | 190 | 0.97 | 0.95 | 0.99 | 0.63 |
| All tests | DR no DMO vs. DM no DR | Microperimetry mesopic | NA | NA | 47 | 190 | 1.00 | 0.99 | 1.00 | 0.63 |
| All tests | DR no DMO vs. DM no DR | Moorfields chart acuity | Near visual acuity | Pelli-Robson contrast sensitivity | 82 | 142 | 0.97 | 0.94 | 0.99 | 0.61 |
| All tests | DR no DMO vs. DM no DR | Moorfields chart acuity | Near visual acuity | Reading index | 29 | 133 | 1.00 | 1.00 | 1.00 | 0.61 |
| All tests | DR no DMO vs. DM no DR | Moorfields chart acuity | Near visual acuity | Smith-Kettlewell low luminance near visual acuity | 39 | 141 | 1.00 | 1.00 | 1.00 | 0.61 |
| All tests | DR no DMO vs. DM no DR | Moorfields chart acuity | Pelli-Robson contrast sensitivity | Reading index | 67 | 133 | 0.99 | 0.97 | 1.00 | 0.61 |
| All tests | DR no DMO vs. DM no DR | Moorfields chart acuity | Pelli-Robson contrast sensitivity | Smith-Kettlewell low luminance near visual acuity | 31 | 141 | 1.00 | 1.00 | 1.00 | 0.62 |
| All tests | DR no DMO vs. DM no DR | Moorfields chart acuity | Reading index | Smith-Kettlewell low luminance near visual acuity | 1 | 132 | 1.00 | 1.00 | 1.00 | 0.62 |
| All tests | DR no DMO vs. DM no DR | Moorfields chart acuity | Reading index | NA | 1 | 133 | 1.00 | 1.00 | 1.00 | 0.61 |
| All tests | DR no DMO vs. DM no DR | Moorfields chart acuity | Smith-Kettlewell low luminance near visual acuity | NA | 58 | 141 | 0.99 | 0.98 | 1.00 | 0.62 |
| All tests | DR no DMO vs. DM no DR | Near visual acuity | Pelli-Robson contrast sensitivity | Reading index | 84 | 194 | 0.96 | 0.94 | 0.98 | 0.58 |
| All tests | DR no DMO vs. DM no DR | Near visual acuity | Pelli-Robson contrast sensitivity | Smith-Kettlewell low luminance near visual acuity | 75 | 205 | 0.98 | 0.96 | 0.99 | 0.60 |
| All tests | DR no DMO vs. DM no DR | Near visual acuity | Reading index | Smith-Kettlewell low luminance near visual acuity | 60 | 194 | 0.99 | 0.98 | 1.00 | 0.59 |
| All tests | DR no DMO vs. DM no DR | Pelli-Robson contrast sensitivity | Reading index | Smith-Kettlewell low luminance near visual acuity | 25 | 193 | 1.00 | 1.00 | 1.00 | 0.59 |
| All tests | DR no DMO vs. DM no DR | Reading index | NA | NA | 88 | 195 | 0.96 | 0.93 | 0.98 | 0.57 |
| All tests | DR with DMO vs. DR no DMO | Distance visual acuity | Low luminance visual acuity | Matrix perimetry | 13 | 143 | 0.97 | 0.95 | 0.99 | 0.79 |
| All tests | DR with DMO vs. DR no DMO | Distance visual acuity | Low luminance visual acuity | Smith-Kettlewell low luminance near visual acuity | 2 | 143 | 0.99 | 0.98 | 1.00 | 0.79 |
| All tests | DR with DMO vs. DR no DMO | Distance visual acuity | Matrix perimetry | Near visual acuity | 11 | 143 | 0.98 | 0.96 | 0.99 | 0.79 |
| All tests | DR with DMO vs. DR no DMO | Distance visual acuity | Matrix perimetry | Pelli-Robson contrast sensitivity | 5 | 142 | 0.99 | 0.97 | 1.00 | 0.80 |
| All tests | DR with DMO vs. DR no DMO | Distance visual acuity | Matrix perimetry | Smith-Kettlewell low luminance near visual acuity | 3 | 143 | 0.99 | 0.98 | 1.00 | 0.79 |
| All tests | DR with DMO vs. DR no DMO | Distance visual acuity | Matrix perimetry | NA | 9 | 143 | 0.98 | 0.96 | 1.00 | 0.78 |
| All tests | DR with DMO vs. DR no DMO | Distance visual acuity | Microperimetry mesopic | Near visual acuity | 10 | 136 | 0.98 | 0.96 | 1.00 | 0.83 |
| All tests | DR with DMO vs. DR no DMO | Distance visual acuity | Microperimetry mesopic | Pelli-Robson contrast sensitivity | 16 | 135 | 0.97 | 0.94 | 0.99 | 0.83 |
| All tests | DR with DMO vs. DR no DMO | Distance visual acuity | Microperimetry mesopic | Reading index | 4 | 127 | 0.99 | 0.98 | 1.00 | 0.82 |
| All tests | DR with DMO vs. DR no DMO | Distance visual acuity | Near visual acuity | Reading index | 21 | 134 | 0.95 | 0.92 | 0.98 | 0.77 |
| All tests | DR with DMO vs. DR no DMO | Distance visual acuity | Near visual acuity | Smith-Kettlewell low luminance near visual acuity | 6 | 143 | 0.98 | 0.97 | 1.00 | 0.80 |
| All tests | DR with DMO vs. DR no DMO | Distance visual acuity | Pelli-Robson contrast sensitivity | Smith-Kettlewell low luminance near visual acuity | 8 | 142 | 0.98 | 0.96 | 1.00 | 0.79 |
| All tests | DR with DMO vs. DR no DMO | Distance visual acuity | Reading index | Smith-Kettlewell low luminance near visual acuity | 20 | 134 | 0.95 | 0.92 | 0.98 | 0.78 |
| All tests | DR with DMO vs. DR no DMO | Distance visual acuity | Smith-Kettlewell low luminance near visual acuity | NA | 23 | 143 | 0.95 | 0.92 | 0.98 | 0.79 |
| All tests | DR with DMO vs. DR no DMO | Low luminance visual acuity | Near visual acuity | Smith-Kettlewell low luminance near visual acuity | 12 | 143 | 0.98 | 0.96 | 0.99 | 0.79 |
| All tests | DR with DMO vs. DR no DMO | Low luminance visual acuity | Pelli-Robson contrast sensitivity | Smith-Kettlewell low luminance near visual acuity | 7 | 142 | 0.98 | 0.96 | 1.00 | 0.76 |
| All tests | DR with DMO vs. DR no DMO | Matrix perimetry | Smith-Kettlewell low luminance near visual acuity | NA | 1 | 143 | 0.99 | 0.99 | 1.00 | 0.61 |
| All tests | DR with DMO vs. DR no DMO | Matrix perimetry | NA | NA | 15 | 143 | 0.97 | 0.95 | 0.99 | 0.61 |
| All tests | DR with DMO vs. DR no DMO | Microperimetry mesopic | Moorfields chart acuity | Near visual acuity | 19 | 95 | 0.95 | 0.92 | 0.99 | 0.87 |
| All tests | DR with DMO vs. DR no DMO | Microperimetry mesopic | Reading index | NA | 18 | 127 | 0.96 | 0.93 | 0.99 | 0.81 |
| All tests | DR with DMO vs. DR no DMO | Moorfields chart acuity | Near visual acuity | Smith-Kettlewell low luminance near visual acuity | 17 | 101 | 0.96 | 0.93 | 1.00 | 0.84 |
| All tests | DR with DMO vs. DR no DMO | Moorfields chart acuity | Pelli-Robson contrast sensitivity | Smith-Kettlewell low luminance near visual acuity | 22 | 101 | 0.95 | 0.91 | 0.99 | 0.85 |
| All tests | DR with DMO vs. DR no DMO | Near visual acuity | Pelli-Robson contrast sensitivity | Smith-Kettlewell low luminance near visual acuity | 14 | 142 | 0.97 | 0.95 | 0.99 | 0.77 |

ESM Table 4. Performance of combinations of visual function tests by task. No perimetry.

| Sample | Task | Var1 | Var2 | Var3 | Rank | N | AUC | AUCLower | AUCUpper | GLMAUC |
| --- | --- | --- | --- | --- | --- | --- | --- | --- | --- | --- |
| No perimetry | DM no DR vs. No DM | Distance visual acuity | Low luminance visual acuity | Moorfields chart acuity | 44 | 1209 | 0.95 | 0.94 | 0.97 | 0.63 |
| No perimetry | DM no DR vs. No DM | Distance visual acuity | Low luminance visual acuity | Near visual acuity | 34 | 1590 | 0.97 | 0.96 | 0.98 | 0.62 |
| No perimetry | DM no DR vs. No DM | Distance visual acuity | Low luminance visual acuity | Pelli-Robson contrast sensitivity | 22 | 1587 | 1.00 | 0.99 | 1.00 | 0.62 |
| No perimetry | DM no DR vs. No DM | Distance visual acuity | Low luminance visual acuity | Reading index | 3 | 1557 | 1.00 | 1.00 | 1.00 | 0.66 |
| No perimetry | DM no DR vs. No DM | Distance visual acuity | Low luminance visual acuity | Smith-Kettlewell low luminance near visual acuity | 33 | 1590 | 0.97 | 0.96 | 0.98 | 0.62 |
| No perimetry | DM no DR vs. No DM | Distance visual acuity | Moorfields chart acuity | Near visual acuity | 26 | 1208 | 0.98 | 0.98 | 0.99 | 0.63 |
| No perimetry | DM no DR vs. No DM | Distance visual acuity | Moorfields chart acuity | Pelli-Robson contrast sensitivity | 28 | 1207 | 0.98 | 0.97 | 0.99 | 0.63 |
| No perimetry | DM no DR vs. No DM | Distance visual acuity | Moorfields chart acuity | Reading index | 6 | 1186 | 1.00 | 1.00 | 1.00 | 0.67 |
| No perimetry | DM no DR vs. No DM | Distance visual acuity | Moorfields chart acuity | NA | 45 | 1211 | 0.95 | 0.94 | 0.96 | 0.63 |
| No perimetry | DM no DR vs. No DM | Distance visual acuity | Near visual acuity | Pelli-Robson contrast sensitivity | 29 | 1588 | 0.98 | 0.97 | 0.98 | 0.62 |
| No perimetry | DM no DR vs. No DM | Distance visual acuity | Near visual acuity | Reading index | 17 | 1557 | 1.00 | 1.00 | 1.00 | 0.66 |
| No perimetry | DM no DR vs. No DM | Distance visual acuity | Near visual acuity | Smith-Kettlewell low luminance near visual acuity | 38 | 1589 | 0.97 | 0.96 | 0.98 | 0.62 |
| No perimetry | DM no DR vs. No DM | Distance visual acuity | Pelli-Robson contrast sensitivity | Reading index | 9 | 1554 | 1.00 | 1.00 | 1.00 | 0.66 |
| No perimetry | DM no DR vs. No DM | Distance visual acuity | Pelli-Robson contrast sensitivity | Smith-Kettlewell low luminance near visual acuity | 23 | 1586 | 0.99 | 0.99 | 1.00 | 0.62 |
| No perimetry | DM no DR vs. No DM | Distance visual acuity | Pelli-Robson contrast sensitivity | NA | 39 | 1589 | 0.97 | 0.96 | 0.98 | 0.62 |
| No perimetry | DM no DR vs. No DM | Distance visual acuity | Reading index | Smith-Kettlewell low luminance near visual acuity | 2 | 1558 | 1.00 | 1.00 | 1.00 | 0.66 |
| No perimetry | DM no DR vs. No DM | Distance visual acuity | Reading index | NA | 16 | 1559 | 1.00 | 1.00 | 1.00 | 0.66 |
| No perimetry | DM no DR vs. No DM | Low luminance visual acuity | Moorfields chart acuity | Reading index | 10 | 1184 | 1.00 | 1.00 | 1.00 | 0.67 |
| No perimetry | DM no DR vs. No DM | Low luminance visual acuity | Moorfields chart acuity | Smith-Kettlewell low luminance near visual acuity | 27 | 1208 | 0.98 | 0.97 | 0.99 | 0.63 |
| No perimetry | DM no DR vs. No DM | Low luminance visual acuity | Near visual acuity | Pelli-Robson contrast sensitivity | 31 | 1586 | 0.98 | 0.97 | 0.98 | 0.62 |
| No perimetry | DM no DR vs. No DM | Low luminance visual acuity | Near visual acuity | Reading index | 7 | 1555 | 1.00 | 1.00 | 1.00 | 0.66 |
| No perimetry | DM no DR vs. No DM | Low luminance visual acuity | Near visual acuity | Smith-Kettlewell low luminance near visual acuity | 36 | 1587 | 0.97 | 0.96 | 0.98 | 0.62 |
| No perimetry | DM no DR vs. No DM | Low luminance visual acuity | Pelli-Robson contrast sensitivity | Reading index | 5 | 1552 | 1.00 | 1.00 | 1.00 | 0.66 |
| No perimetry | DM no DR vs. No DM | Low luminance visual acuity | Pelli-Robson contrast sensitivity | Smith-Kettlewell low luminance near visual acuity | 24 | 1584 | 0.99 | 0.99 | 0.99 | 0.62 |
| No perimetry | DM no DR vs. No DM | Low luminance visual acuity | Pelli-Robson contrast sensitivity | NA | 40 | 1587 | 0.96 | 0.95 | 0.97 | 0.62 |
| No perimetry | DM no DR vs. No DM | Low luminance visual acuity | Reading index | Smith-Kettlewell low luminance near visual acuity | 1 | 1556 | 1.00 | 1.00 | 1.00 | 0.66 |
| No perimetry | DM no DR vs. No DM | Low luminance visual acuity | Reading index | NA | 11 | 1557 | 1.00 | 1.00 | 1.00 | 0.66 |
| No perimetry | DM no DR vs. No DM | Low luminance visual acuity | Smith-Kettlewell low luminance near visual acuity | NA | 43 | 1590 | 0.95 | 0.94 | 0.96 | 0.62 |
| No perimetry | DM no DR vs. No DM | Moorfields chart acuity | Near visual acuity | Pelli-Robson contrast sensitivity | 37 | 1206 | 0.97 | 0.96 | 0.98 | 0.62 |
| No perimetry | DM no DR vs. No DM | Moorfields chart acuity | Near visual acuity | Reading index | 20 | 1184 | 1.00 | 1.00 | 1.00 | 0.67 |
| No perimetry | DM no DR vs. No DM | Moorfields chart acuity | Near visual acuity | Smith-Kettlewell low luminance near visual acuity | 35 | 1207 | 0.97 | 0.96 | 0.98 | 0.62 |
| No perimetry | DM no DR vs. No DM | Moorfields chart acuity | Near visual acuity | NA | 42 | 1208 | 0.96 | 0.94 | 0.97 | 0.62 |
| No perimetry | DM no DR vs. No DM | Moorfields chart acuity | Pelli-Robson contrast sensitivity | Reading index | 12 | 1183 | 1.00 | 1.00 | 1.00 | 0.67 |
| No perimetry | DM no DR vs. No DM | Moorfields chart acuity | Pelli-Robson contrast sensitivity | Smith-Kettlewell low luminance near visual acuity | 32 | 1206 | 0.97 | 0.96 | 0.98 | 0.62 |
| No perimetry | DM no DR vs. No DM | Moorfields chart acuity | Reading index | Smith-Kettlewell low luminance near visual acuity | 4 | 1185 | 1.00 | 1.00 | 1.00 | 0.67 |
| No perimetry | DM no DR vs. No DM | Moorfields chart acuity | Reading index | NA | 14 | 1186 | 1.00 | 1.00 | 1.00 | 0.67 |
| No perimetry | DM no DR vs. No DM | Near visual acuity | Pelli-Robson contrast sensitivity | Reading index | 19 | 1553 | 1.00 | 1.00 | 1.00 | 0.65 |
| No perimetry | DM no DR vs. No DM | Near visual acuity | Pelli-Robson contrast sensitivity | Smith-Kettlewell low luminance near visual acuity | 41 | 1585 | 0.96 | 0.95 | 0.97 | 0.62 |
| No perimetry | DM no DR vs. No DM | Near visual acuity | Reading index | Smith-Kettlewell low luminance near visual acuity | 8 | 1556 | 1.00 | 1.00 | 1.00 | 0.65 |
| No perimetry | DM no DR vs. No DM | Near visual acuity | Reading index | NA | 18 | 1557 | 1.00 | 1.00 | 1.00 | 0.65 |
| No perimetry | DM no DR vs. No DM | Pelli-Robson contrast sensitivity | Reading index | Smith-Kettlewell low luminance near visual acuity | 15 | 1553 | 1.00 | 1.00 | 1.00 | 0.65 |
| No perimetry | DM no DR vs. No DM | Pelli-Robson contrast sensitivity | Reading index | NA | 21 | 1554 | 1.00 | 1.00 | 1.00 | 0.65 |
| No perimetry | DM no DR vs. No DM | Pelli-Robson contrast sensitivity | Smith-Kettlewell low luminance near visual acuity | NA | 30 | 1586 | 0.98 | 0.97 | 0.98 | 0.62 |
| No perimetry | DM no DR vs. No DM | Reading index | Smith-Kettlewell low luminance near visual acuity | NA | 13 | 1558 | 1.00 | 1.00 | 1.00 | 0.66 |
| No perimetry | DM no DR vs. No DM | Reading index | NA | NA | 25 | 1559 | 0.99 | 0.99 | 0.99 | 0.65 |
| No perimetry | DR no DMO vs. DM no DR | Distance visual acuity | Low luminance visual acuity | Pelli-Robson contrast sensitivity | 39 | 490 | 0.96 | 0.94 | 0.97 | 0.59 |
| No perimetry | DR no DMO vs. DM no DR | Distance visual acuity | Low luminance visual acuity | Reading index | 14 | 440 | 0.99 | 0.99 | 1.00 | 0.58 |
| No perimetry | DR no DMO vs. DM no DR | Distance visual acuity | Low luminance visual acuity | Smith-Kettlewell low luminance near visual acuity | 6 | 493 | 1.00 | 0.99 | 1.00 | 0.59 |
| No perimetry | DR no DMO vs. DM no DR | Distance visual acuity | Moorfields chart acuity | Pelli-Robson contrast sensitivity | 36 | 347 | 0.96 | 0.95 | 0.98 | 0.58 |
| No perimetry | DR no DMO vs. DM no DR | Distance visual acuity | Moorfields chart acuity | Reading index | 29 | 305 | 0.98 | 0.97 | 0.99 | 0.61 |
| No perimetry | DR no DMO vs. DM no DR | Distance visual acuity | Moorfields chart acuity | Smith-Kettlewell low luminance near visual acuity | 18 | 348 | 0.99 | 0.99 | 1.00 | 0.61 |
| No perimetry | DR no DMO vs. DM no DR | Distance visual acuity | Moorfields chart acuity | NA | 32 | 349 | 0.97 | 0.96 | 0.99 | 0.56 |
| No perimetry | DR no DMO vs. DM no DR | Distance visual acuity | Near visual acuity | Reading index | 5 | 439 | 1.00 | 1.00 | 1.00 | 0.58 |
| No perimetry | DR no DMO vs. DM no DR | Distance visual acuity | Near visual acuity | Smith-Kettlewell low luminance near visual acuity | 26 | 491 | 0.99 | 0.98 | 0.99 | 0.59 |
| No perimetry | DR no DMO vs. DM no DR | Distance visual acuity | Pelli-Robson contrast sensitivity | Reading index | 17 | 437 | 0.99 | 0.99 | 1.00 | 0.60 |
| No perimetry | DR no DMO vs. DM no DR | Distance visual acuity | Pelli-Robson contrast sensitivity | Smith-Kettlewell low luminance near visual acuity | 20 | 489 | 0.99 | 0.99 | 1.00 | 0.60 |
| No perimetry | DR no DMO vs. DM no DR | Distance visual acuity | Reading index | Smith-Kettlewell low luminance near visual acuity | 1 | 439 | 1.00 | 1.00 | 1.00 | 0.60 |
| No perimetry | DR no DMO vs. DM no DR | Distance visual acuity | Reading index | NA | 13 | 440 | 0.99 | 0.99 | 1.00 | 0.58 |
| No perimetry | DR no DMO vs. DM no DR | Distance visual acuity | Smith-Kettlewell low luminance near visual acuity | NA | 37 | 493 | 0.96 | 0.95 | 0.98 | 0.59 |
| No perimetry | DR no DMO vs. DM no DR | Low luminance visual acuity | Moorfields chart acuity | Reading index | 9 | 305 | 1.00 | 0.99 | 1.00 | 0.61 |
| No perimetry | DR no DMO vs. DM no DR | Low luminance visual acuity | Moorfields chart acuity | Smith-Kettlewell low luminance near visual acuity | 11 | 348 | 0.99 | 0.99 | 1.00 | 0.61 |
| No perimetry | DR no DMO vs. DM no DR | Low luminance visual acuity | Near visual acuity | Pelli-Robson contrast sensitivity | 35 | 490 | 0.97 | 0.95 | 0.98 | 0.60 |
| No perimetry | DR no DMO vs. DM no DR | Low luminance visual acuity | Near visual acuity | Reading index | 4 | 439 | 1.00 | 1.00 | 1.00 | 0.58 |
| No perimetry | DR no DMO vs. DM no DR | Low luminance visual acuity | Near visual acuity | Smith-Kettlewell low luminance near visual acuity | 33 | 491 | 0.97 | 0.96 | 0.98 | 0.59 |
| No perimetry | DR no DMO vs. DM no DR | Low luminance visual acuity | Near visual acuity | NA | 38 | 492 | 0.96 | 0.94 | 0.97 | 0.57 |
| No perimetry | DR no DMO vs. DM no DR | Low luminance visual acuity | Pelli-Robson contrast sensitivity | Reading index | 19 | 437 | 0.99 | 0.99 | 1.00 | 0.60 |
| No perimetry | DR no DMO vs. DM no DR | Low luminance visual acuity | Pelli-Robson contrast sensitivity | Smith-Kettlewell low luminance near visual acuity | 12 | 489 | 0.99 | 0.99 | 1.00 | 0.61 |
| No perimetry | DR no DMO vs. DM no DR | Low luminance visual acuity | Reading index | Smith-Kettlewell low luminance near visual acuity | 3 | 439 | 1.00 | 1.00 | 1.00 | 0.60 |
| No perimetry | DR no DMO vs. DM no DR | Low luminance visual acuity | Reading index | NA | 15 | 440 | 0.99 | 0.99 | 1.00 | 0.58 |
| No perimetry | DR no DMO vs. DM no DR | Moorfields chart acuity | Near visual acuity | Pelli-Robson contrast sensitivity | 40 | 347 | 0.96 | 0.94 | 0.98 | 0.61 |
| No perimetry | DR no DMO vs. DM no DR | Moorfields chart acuity | Near visual acuity | Reading index | 16 | 304 | 0.99 | 0.99 | 1.00 | 0.63 |
| No perimetry | DR no DMO vs. DM no DR | Moorfields chart acuity | Near visual acuity | NA | 31 | 347 | 0.98 | 0.96 | 0.99 | 0.60 |
| No perimetry | DR no DMO vs. DM no DR | Moorfields chart acuity | Pelli-Robson contrast sensitivity | Reading index | 22 | 304 | 0.99 | 0.98 | 1.00 | 0.62 |
| No perimetry | DR no DMO vs. DM no DR | Moorfields chart acuity | Pelli-Robson contrast sensitivity | Smith-Kettlewell low luminance near visual acuity | 21 | 346 | 0.99 | 0.98 | 1.00 | 0.61 |
| No perimetry | DR no DMO vs. DM no DR | Moorfields chart acuity | Pelli-Robson contrast sensitivity | NA | 34 | 347 | 0.97 | 0.95 | 0.98 | 0.59 |
| No perimetry | DR no DMO vs. DM no DR | Moorfields chart acuity | Reading index | Smith-Kettlewell low luminance near visual acuity | 2 | 304 | 1.00 | 1.00 | 1.00 | 0.64 |
| No perimetry | DR no DMO vs. DM no DR | Moorfields chart acuity | Reading index | NA | 25 | 305 | 0.99 | 0.98 | 1.00 | 0.61 |
| No perimetry | DR no DMO vs. DM no DR | Moorfields chart acuity | Smith-Kettlewell low luminance near visual acuity | NA | 27 | 348 | 0.99 | 0.98 | 1.00 | 0.61 |
| No perimetry | DR no DMO vs. DM no DR | Near visual acuity | Pelli-Robson contrast sensitivity | Reading index | 23 | 437 | 0.99 | 0.98 | 1.00 | 0.60 |
| No perimetry | DR no DMO vs. DM no DR | Near visual acuity | Pelli-Robson contrast sensitivity | Smith-Kettlewell low luminance near visual acuity | 30 | 489 | 0.98 | 0.97 | 0.99 | 0.61 |
| No perimetry | DR no DMO vs. DM no DR | Near visual acuity | Reading index | Smith-Kettlewell low luminance near visual acuity | 7 | 438 | 1.00 | 0.99 | 1.00 | 0.60 |
| No perimetry | DR no DMO vs. DM no DR | Near visual acuity | Reading index | NA | 24 | 439 | 0.99 | 0.98 | 1.00 | 0.58 |
| No perimetry | DR no DMO vs. DM no DR | Pelli-Robson contrast sensitivity | Reading index | Smith-Kettlewell low luminance near visual acuity | 10 | 436 | 1.00 | 0.99 | 1.00 | 0.60 |
| No perimetry | DR no DMO vs. DM no DR | Pelli-Robson contrast sensitivity | Reading index | NA | 28 | 437 | 0.98 | 0.97 | 0.99 | 0.59 |
| No perimetry | DR no DMO vs. DM no DR | Reading index | Smith-Kettlewell low luminance near visual acuity | NA | 8 | 439 | 1.00 | 0.99 | 1.00 | 0.60 |
| No perimetry | DR no DMO vs. DM no DR | Reading index | NA | NA | 41 | 440 | 0.96 | 0.94 | 0.97 | 0.58 |
| No perimetry | DR with DMO vs. DR no DMO | Distance visual acuity | Low luminance visual acuity | Near visual acuity | 30 | 306 | 0.97 | 0.95 | 0.98 | 0.81 |
| No perimetry | DR with DMO vs. DR no DMO | Distance visual acuity | Low luminance visual acuity | Reading index | 15 | 275 | 0.99 | 0.98 | 1.00 | 0.79 |
| No perimetry | DR with DMO vs. DR no DMO | Distance visual acuity | Moorfields chart acuity | Reading index | 20 | 186 | 0.98 | 0.97 | 1.00 | 0.85 |
| No perimetry | DR with DMO vs. DR no DMO | Distance visual acuity | Moorfields chart acuity | Smith-Kettlewell low luminance near visual acuity | 32 | 214 | 0.97 | 0.94 | 0.99 | 0.87 |
| No perimetry | DR with DMO vs. DR no DMO | Distance visual acuity | Near visual acuity | Reading index | 3 | 275 | 1.00 | 1.00 | 1.00 | 0.79 |
| No perimetry | DR with DMO vs. DR no DMO | Distance visual acuity | Pelli-Robson contrast sensitivity | Reading index | 21 | 273 | 0.98 | 0.97 | 0.99 | 0.79 |
| No perimetry | DR with DMO vs. DR no DMO | Distance visual acuity | Reading index | Smith-Kettlewell low luminance near visual acuity | 1 | 275 | 1.00 | 1.00 | 1.00 | 0.80 |
| No perimetry | DR with DMO vs. DR no DMO | Distance visual acuity | Reading index | NA | 28 | 275 | 0.97 | 0.96 | 0.99 | 0.78 |
| No perimetry | DR with DMO vs. DR no DMO | Low luminance visual acuity | Moorfields chart acuity | Near visual acuity | 36 | 214 | 0.95 | 0.93 | 0.98 | 0.86 |
| No perimetry | DR with DMO vs. DR no DMO | Low luminance visual acuity | Moorfields chart acuity | Pelli-Robson contrast sensitivity | 26 | 214 | 0.98 | 0.96 | 0.99 | 0.87 |
| No perimetry | DR with DMO vs. DR no DMO | Low luminance visual acuity | Moorfields chart acuity | Reading index | 29 | 186 | 0.97 | 0.95 | 0.99 | 0.85 |
| No perimetry | DR with DMO vs. DR no DMO | Low luminance visual acuity | Near visual acuity | Pelli-Robson contrast sensitivity | 31 | 304 | 0.97 | 0.95 | 0.98 | 0.80 |
| No perimetry | DR with DMO vs. DR no DMO | Low luminance visual acuity | Near visual acuity | Reading index | 9 | 275 | 0.99 | 0.99 | 1.00 | 0.80 |
| No perimetry | DR with DMO vs. DR no DMO | Low luminance visual acuity | Near visual acuity | Smith-Kettlewell low luminance near visual acuity | 16 | 306 | 0.99 | 0.98 | 1.00 | 0.82 |
| No perimetry | DR with DMO vs. DR no DMO | Low luminance visual acuity | Near visual acuity | NA | 37 | 306 | 0.95 | 0.93 | 0.97 | 0.81 |
| No perimetry | DR with DMO vs. DR no DMO | Low luminance visual acuity | Pelli-Robson contrast sensitivity | Reading index | 13 | 273 | 0.99 | 0.98 | 1.00 | 0.78 |
| No perimetry | DR with DMO vs. DR no DMO | Low luminance visual acuity | Pelli-Robson contrast sensitivity | Smith-Kettlewell low luminance near visual acuity | 25 | 304 | 0.98 | 0.96 | 0.99 | 0.79 |
| No perimetry | DR with DMO vs. DR no DMO | Low luminance visual acuity | Reading index | Smith-Kettlewell low luminance near visual acuity | 2 | 275 | 1.00 | 1.00 | 1.00 | 0.79 |
| No perimetry | DR with DMO vs. DR no DMO | Low luminance visual acuity | Reading index | NA | 12 | 275 | 0.99 | 0.98 | 1.00 | 0.79 |
| No perimetry | DR with DMO vs. DR no DMO | Moorfields chart acuity | Near visual acuity | Pelli-Robson contrast sensitivity | 22 | 214 | 0.98 | 0.97 | 0.99 | 0.86 |
| No perimetry | DR with DMO vs. DR no DMO | Moorfields chart acuity | Near visual acuity | Reading index | 8 | 186 | 1.00 | 0.99 | 1.00 | 0.85 |
| No perimetry | DR with DMO vs. DR no DMO | Moorfields chart acuity | Near visual acuity | Smith-Kettlewell low luminance near visual acuity | 10 | 214 | 0.99 | 0.99 | 1.00 | 0.87 |
| No perimetry | DR with DMO vs. DR no DMO | Moorfields chart acuity | Pelli-Robson contrast sensitivity | Reading index | 14 | 186 | 0.99 | 0.98 | 1.00 | 0.85 |
| No perimetry | DR with DMO vs. DR no DMO | Moorfields chart acuity | Pelli-Robson contrast sensitivity | Smith-Kettlewell low luminance near visual acuity | 17 | 214 | 0.99 | 0.98 | 1.00 | 0.87 |
| No perimetry | DR with DMO vs. DR no DMO | Moorfields chart acuity | Pelli-Robson contrast sensitivity | NA | 34 | 214 | 0.96 | 0.94 | 0.98 | 0.86 |
| No perimetry | DR with DMO vs. DR no DMO | Moorfields chart acuity | Reading index | Smith-Kettlewell low luminance near visual acuity | 11 | 186 | 0.99 | 0.99 | 1.00 | 0.85 |
| No perimetry | DR with DMO vs. DR no DMO | Moorfields chart acuity | Reading index | NA | 18 | 186 | 0.99 | 0.98 | 1.00 | 0.85 |
| No perimetry | DR with DMO vs. DR no DMO | Moorfields chart acuity | Smith-Kettlewell low luminance near visual acuity | NA | 24 | 214 | 0.98 | 0.96 | 0.99 | 0.87 |
| No perimetry | DR with DMO vs. DR no DMO | Near visual acuity | Pelli-Robson contrast sensitivity | Reading index | 6 | 273 | 1.00 | 0.99 | 1.00 | 0.78 |
| No perimetry | DR with DMO vs. DR no DMO | Near visual acuity | Pelli-Robson contrast sensitivity | Smith-Kettlewell low luminance near visual acuity | 35 | 304 | 0.96 | 0.94 | 0.98 | 0.81 |
| No perimetry | DR with DMO vs. DR no DMO | Near visual acuity | Reading index | Smith-Kettlewell low luminance near visual acuity | 7 | 275 | 1.00 | 0.99 | 1.00 | 0.79 |
| No perimetry | DR with DMO vs. DR no DMO | Near visual acuity | Reading index | NA | 19 | 275 | 0.99 | 0.98 | 1.00 | 0.77 |
| No perimetry | DR with DMO vs. DR no DMO | Pelli-Robson contrast sensitivity | Reading index | Smith-Kettlewell low luminance near visual acuity | 5 | 273 | 1.00 | 0.99 | 1.00 | 0.71 |
| No perimetry | DR with DMO vs. DR no DMO | Pelli-Robson contrast sensitivity | Reading index | NA | 27 | 273 | 0.98 | 0.96 | 0.99 | 0.70 |
| No perimetry | DR with DMO vs. DR no DMO | Pelli-Robson contrast sensitivity | Smith-Kettlewell low luminance near visual acuity | NA | 33 | 304 | 0.96 | 0.94 | 0.98 | 0.72 |
| No perimetry | DR with DMO vs. DR no DMO | Reading index | Smith-Kettlewell low luminance near visual acuity | NA | 4 | 275 | 1.00 | 0.99 | 1.00 | 0.67 |
| No perimetry | DR with DMO vs. DR no DMO | Reading index | NA | NA | 23 | 275 | 0.98 | 0.97 | 0.99 | 0.64 |
